# Evolving Nature of Tuberculosis in the Post-pandemic World

**DOI:** 10.7759/cureus.98349

**Published:** 2025-12-02

**Authors:** Tamanna Bordoloi, Kiran Bala, Jaya Biswas, Neha Nityadarshini, Raunak Bir, Pawan Tiwari, Saurabh Mittal, Praveen Bharti, Anant Mohan, Urvashi Singh

**Affiliations:** 1 Department of Microbiology, All India Institute of Medical Sciences, New Delhi, New Delhi, IND; 2 Department of Microbiology, Employee's State Insurance Corporation (ESIC) Medical College and Hospital, Faridabad, IND; 3 Department of Pulmonary, Critical Care, and Sleep Medicine, All India Institute of Medical Sciences, New Delhi, New Delhi, IND; 4 Department of Medicine, Maulana Azad Medical College, New Delhi, IND

**Keywords:** covid-19, covid-19 vaccination, genexpert, immune response, tuberculosis

## Abstract

Introduction

Tuberculosis (TB) and COVID-19 are highly transmissible respiratory diseases. The sequel of development of active TB after COVID-19 disease has been hypothesized to increase due to many reasons. We aimed to explore the association between COVID-19 infection, COVID-19 vaccination, and the development of active TB and navigate any existing patterns if present.

Materials and methods

This prospective observational study was conducted over a span of one year in two hospitals. A total of 200 patients with suspected TB were recruited and divided into two groups: Group A (n=100) had previous infection with COVID-19, and Group B (n=100) had no previous COVID-19 disease. Diagnosis of TB was done from both pulmonary and extrapulmonary samples by microscopy using the Ziehl-Neelsen (ZN) technique for acid-fast bacilli, culture using the Mycobacteria Growth Indicator Tube-960 (MGIT-960) (Becton, Dickinson and Company, Sparks, Maryland, United States), and molecular assays such as the GeneXpert® MTB/RIF (Cepheid, Sunnyvale, California, United States). Epidemiological data, clinical picture, and their pattern of sequence of TB after COVID-19 vaccination and COVID-19 disease were investigated.

Results* *

In Group A, 37 (37%) were females with a mean age of 39.4 years, and 63 (63%) were males with a mean age of 41.9 years. In Group B, 45 (45%) were females, and 55 (55%) were males, with mean ages of 41.02 years and 44.16 years, respectively. Confirmation of TB cases was done using ZN staining, MGIT-960, and GeneXpert® MTB/RIF assay. Active TB infection was identified in 14 (14%) participants in Group A, of whom 11 (78.5%) were COVID-19 vaccinated, and in 19 (19%) participants in Group B, of whom 14 (73.6%) were vaccinated. Covishield was the predominant vaccine received. Most of the patients had received both doses of the vaccine. Logistic regression analysis was performed, which showed that vaccinated individuals had a lower odds of TB infection than unvaccinated individuals in both groups. However, the findings were not statistically significant (p>0.05).

Conclusion

No association between COVID-19 vaccination and the subsequent development of active TB was seen.

## Introduction

Tuberculosis (TB) and COVID-19 are highly transmissible respiratory diseases that pose a risk to public health. Both diseases have a similar route of transmission, and dysregulation of the immune system is a known feature in both [1]. Both diseases are spread through droplets and contact with an infected person. Symptoms such as fever, cough, vomiting, and shortness of breath may be present in patients with both diseases. COVID-19 is more severe with greater chances of mortality in the elderly and also in people with underlying diseases. Coinfections are quite common in COVID-19, and a major cause of concern in an endemic country such as India is TB. Both diseases share some common determinants, such as overcrowding, diabetes, hypertension, and respiratory diseases, including chronic obstructive pulmonary disease (COPD). Overcrowding is a risk factor for diseases with airborne transmission, which is why overcrowded cities in India have reported the highest cases of COVID-19. Diabetes also contributes as a major comorbid condition for both diseases [2]. It is more difficult to diagnose and treat COVID-19 when it coexists with TB infection. This also contributes to the increased risk of death and non-recovery to a significant extent [3]. Even though TB is a curable disease, 10.8 million new cases are reported each year, mostly in low- and middle-income nations. Multidrug-resistant (MDR) TB is a major cause of death, affecting an estimated 400,000 people annually.

In 2023, TB reclaimed its position as the leading infectious disease responsible for mortality, accounting for an estimated 1.25 million deaths, followed by COVID-19. There may be an additional 6.3 million TB cases and 1.4 million TB deaths between 2020 and 2025 as a consequence of the aftermath of the COVID-19 pandemic [4,5]. The COVID-19 pandemic has caused the current goal of TB elimination by 2025 to be disrupted, although efforts are underway to get back on track [6-9].

It has been postulated that a variety of reasons can lead to an increase in the incidence of active TB following COVID-19 disease. Our goal was to evaluate how COVID-19 disease and COVID-19 response strategies, such as COVID-19 vaccination, affected TB. Therefore, the objective of this study is to determine whether prior COVID-19 infection and COVID-19 vaccination status are associated with the development of active TB.

## Materials and methods

Study design and participants

This prospective observational study was conducted in the Department of Microbiology in collaboration with the Department of Pulmonary Medicine and Sleep Disorders, Department of Medicine, Department of Orthopedics, and Department of Gastroenterology in All India Institute of Medical Sciences, New Delhi, India. Patients were also enrolled from the Department of Medicine, Lok Nayak Hospital, New Delhi, India. The study was conducted in a span of one year and was approved by the Institute Ethics Committee of the All India Institute of Medical Sciences, New Delhi (approval number: IEC-740/12.11.2021, RP-13/2021/25.11.2021). Suspected pulmonary and extrapulmonary TB cases were enrolled based on the clinical presentation and consent to participate.

The study included individuals who were above 13 years of age and those who had active TB with or without COVID-19. Patients not giving consent, patients who had received anti-TB therapy within the last six months, pregnant women, and human immunodeficiency virus (HIV)-infected patients were excluded from the study. Eligible participants were enrolled until the target sample size was achieved.

Study Population

The study population comprised 200 suspected pulmonary TB cases attending the outpatient/inpatient department in the Department of Pulmonary Medicine and Sleep Disorders, Department of Medicine, Department of Orthopedics, and Department of Gastroenterology in All India Institute of Medical Sciences, New Delhi, India, and also the Department of Medicine in Lok Nayak Hospital, New Delhi, India.

Study Design

**Figure 1:**
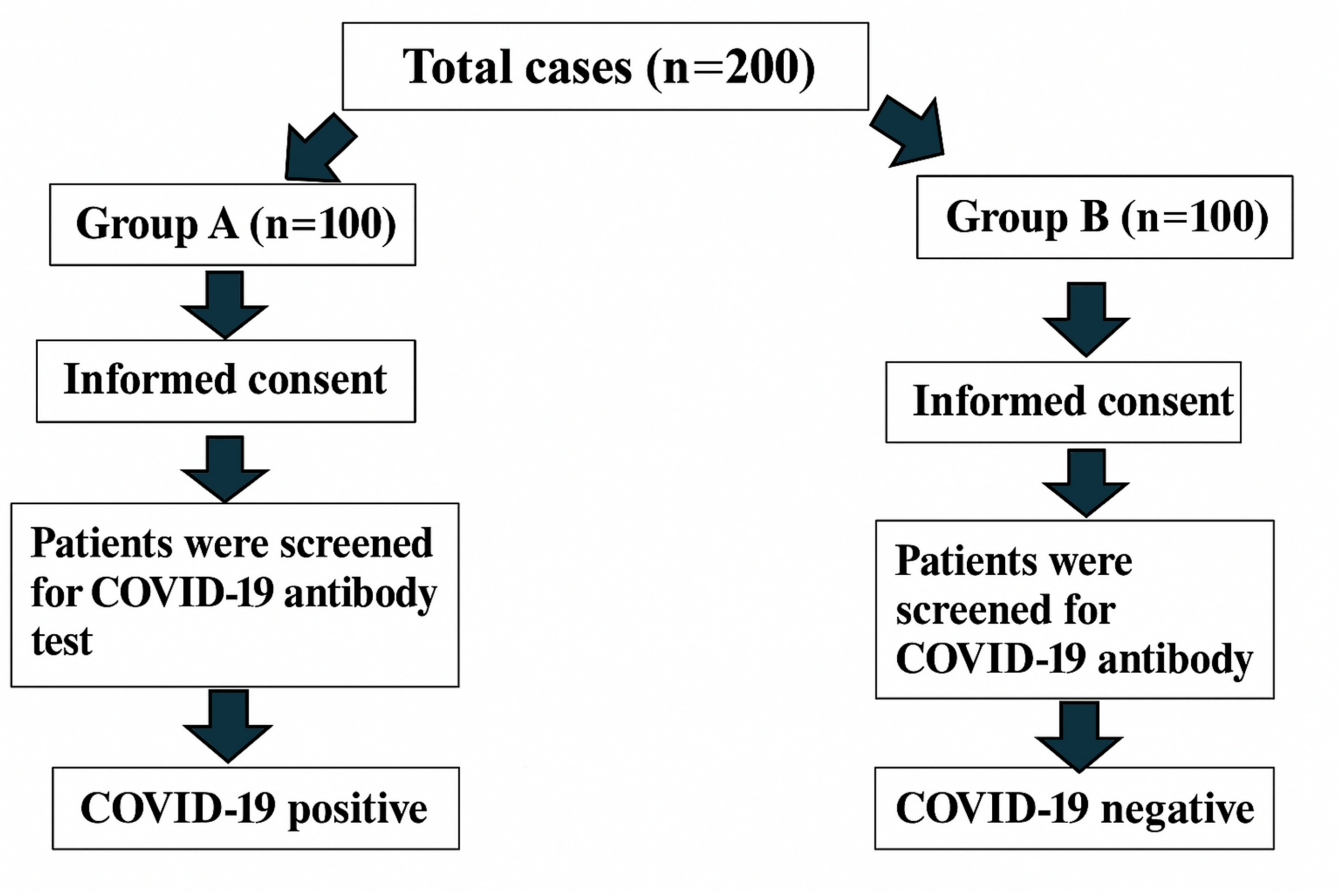
Identification of cases and controls The flowchart shows the steps from informed consent to grouping of patients based on COVID-19 antibody testing results. Group A: COVID-19 positive; Group B: COVID-19 negative

**Figure 2:**
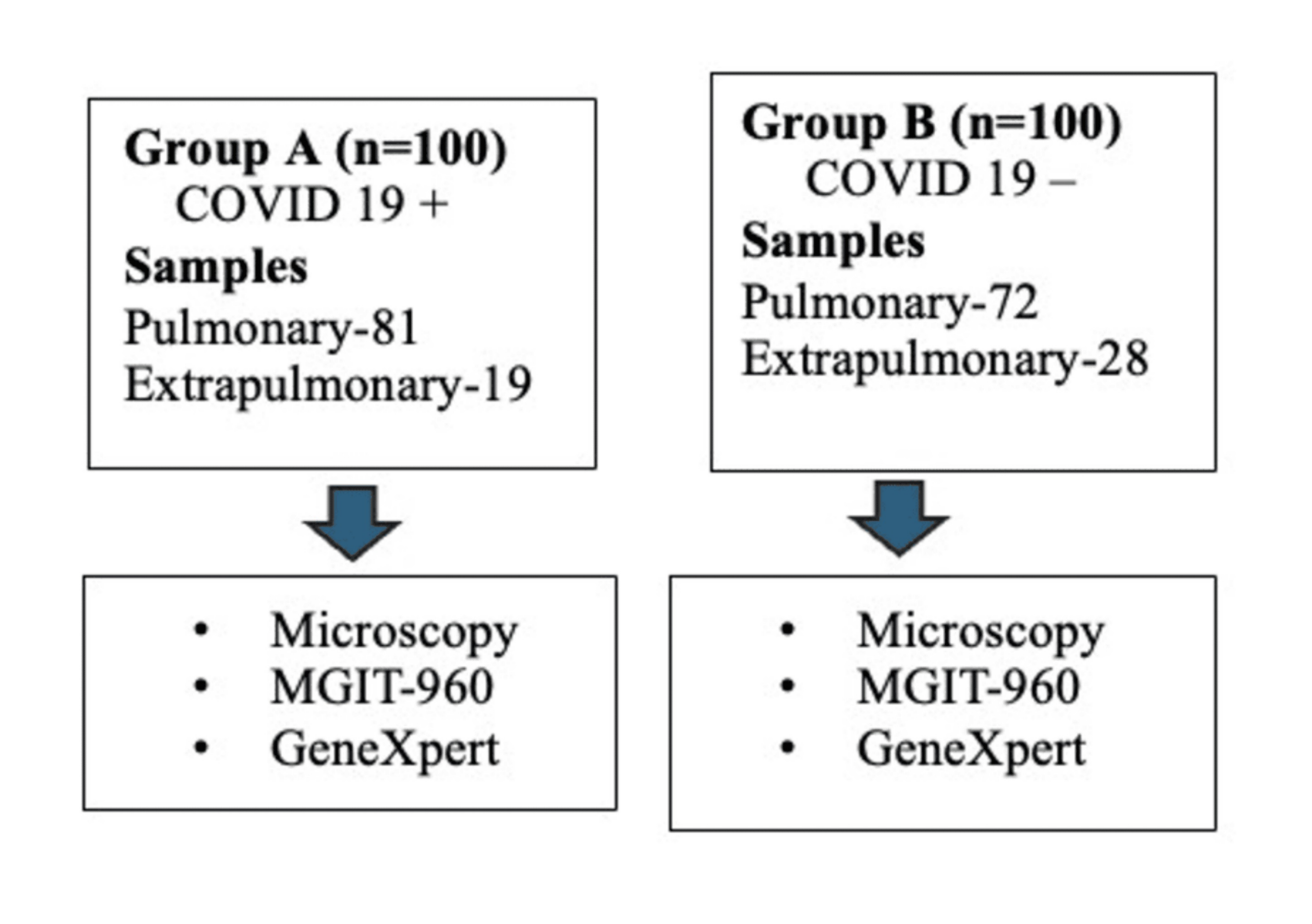
Workflow for the identification of active TB The workflow depicts the sample types (pulmonary vs. extrapulmonary) and diagnostic methods used for TB identification in both groups. Group A: COVID-19 positive; Group B: COVID-19 negative TB: tuberculosis; MGIT-960: Mycobacteria Growth Indicator Tube-960

The study was conducted over a span of one year (2021-2022). A total of 200 patients with suspected TB were recruited and divided into two groups: Group A (n=100) had previous infection with COVID-19, and Group B (n=100) had no previous COVID-19 disease. Diagnosis of TB was done from both pulmonary and extrapulmonary samples by microscopy using the Ziehl-Neelsen (ZN) technique for acid-fast bacilli (AFB), culture using the Mycobacteria Growth Indicator Tube-960 (MGIT-960) (Becton, Dickinson and Company, Sparks, Maryland, United States), and molecular assays such as the GeneXpert® MTB/RIF (Cepheid, Sunnyvale, California, United States). Epidemiological data, clinical picture, and their pattern of sequence of TB after COVID-19 vaccination and COVID-19 disease were investigated. The workflow is depicted in Figures 1-2.

Diagnostic definition of TB disease

Active TB disease was defined strictly on the basis of microbiological and clinical confirmation. Together with a clinical assessment by a pulmonologist, only patients who had one or more of the following were classified as having active TB disease: sputum smear positivity, cartridge-based nucleic acid amplification test (CBNAAT)/GeneXpert positivity, culture confirmation, and radiographic findings consistent with TB. Latent TB infection was not assessed in this study as tuberculin skin test (TST)/interferon-gamma release assay (IGRA) testing were not performed. Therefore, individuals without diagnostic confirmation were not classified as active TB disease [10,11].

TB case definitions

Based on clinical, radiological, and microbiological criteria, each patient was allocated to one of the diagnostic categories as defined by the World Health Organization and the National TB Elimination Programme (NTEP) [10,11].

Confirmed TB

It is defined as a clinical presentation compatible with TB in which *Mycobacterium tuberculosis *complex is cultured from any clinical specimen, AFB are demonstrated by microscopy, or granulomatous inflammation is seen histologically in the absence of non-TB mycobacteria growth.

Non-TB

It is defined as no microbiological or radiological evidence of active TB disease, with or without any other alternative diagnoses identified during the patient's follow-up.

Diagnostic procedures

Sputum and extrapulmonary specimens were processed according to the NTEP diagnostic standards. ZN smear microscopy was performed following the NTEP protocol for staining and grading of AFB [12]. Culture and drug susceptibility testing were carried out using the MGIT-960 system in accordance with the NTEP diagnostic guidelines [13]. CBNAAT using the GeneXpert® MTB/RIF assay was performed as per NTEP recommendations [14].

Lab diagnosis

Microscopy

Decontaminated samples were stained using the ZN technique. AFB positivity confirmed TB diagnosis [12].

Culture

Concentrated sediment was inoculated in MGIT-960 and incubated at 37°C. The MGIT-960 medium is an enriched Middlebrook 7H9 base. The MGIT-960 contains an oxygen-quenched fluorochrome (tris(4,7-diphenyl-1,10-phenanthroline)ruthenium chloride pentahydrate) embedded in silicon at the bottom of the tube, which detects bacterial growth by fluorescence caused by oxygen depletion. Contamination was ruled out by culture on blood agar, and sterility and positivity were checked by ZN smear microscopy [13].

Molecular Diagnosis

The GeneXpert® MTB/RIF assay was used, which uses hemi-nested real-time polymerase chain reaction (RT-PCR) to amplify an MTB-specific sequence of the rpoB gene. To determine rifampicin resistance, the rpoB gene is probed with molecular beacons within the rifampicin resistance-determining region. It is a fully automated assay and runs on the GeneXpert platform. It uses a disposable plastic cartridge with all required reagents. The cartridge is inserted into the test device, and the automatically generated results are read after 90 minutes [14].

Determination of Prior COVID-19 Infection

Prior COVID-19 infection status was confirmed using documentation in hospital electronic records, including positive RT-PCR results or rapid antigen test positivity. Vaccination status was confirmed through government records or vaccination certificates. Self-reported history without medical documentation was not accepted for classification.

Control of Confounding Variables

We collected information on age, sex, symptoms, comorbidities, and vaccination status for all participants. These factors were compared between the COVID-19-positive and COVID-19-negative groups. Because the sample size was small, we were not able to adjust for all confounders using multivariable analysis. 

Statistical analysis

R version 4.0.2 (R Foundation for Statistical Computing, Vienna, Austria) was used to conduct statistical analyses. Clinical and demographic features were compiled using descriptive statistics. Continuous variables were described using the arithmetic mean, whereas categorical variables were displayed as counts and percentages. A binary logistic regression analysis was used to evaluate the relationship between the occurrence of TB infection and COVID-19 vaccination status. To ascertain the degree and statistical significance of correlations, logistic regression models calculated odds ratios (ORs) with 95% CIs and p-values. Statistical significance was defined as a p-value of less than 0.05. All statistical tests were two-tailed.

## Results

Demographic details

In Group A, 37 (37%) were females with a mean age of 39.4 years, and 63 (63%) were males with a mean age of 41.9 years. In Group B, 45 (45%) were females, and 55 (55%) were males, with mean ages of 41.02 years and 44.16 years, respectively. Active TB infection was identified in 14 (14%) participants in Group A, of whom 11 (78.5%) were COVID-19 vaccinated, and in 19 (19%) participants in Group B, of whom 14 (73.6%) were vaccinated. Among the cases of active TB infection in Group A, the mean age was 40 years for males, and it was 49 years for females. In Group B, mean ages were 21 years and 11.3 years, respectively, for male and female cases. 

Table 1 presents a comparison of demographic and clinical characteristics between Group A and Group B. No statistically significant differences were observed between the groups across most variables. The distribution of sex (p=0.47), employment status (p=0.77), fever (p=0.55), cough (p=0.11), loss of appetite (p=0.20), bronchial asthma (p=0.61), hypertension (p=0.54), HIV status (p=0.48), and chronic kidney disease (p=0.61) were comparable between the two groups. However, past history of TB (p=0.07) and loss of weight (p=0.09) showed near-significant differences with a higher proportion of TB history in Group B (38, 38%) compared to Group A (25, 25%). More individuals reporting weight loss were present in Group A (53, 53%) compared to Group B (40, 40%). These trends may suggest potential associations that merit further investigation in larger cohorts.

**Table 1:** Baseline characteristics of cases and controls This table summarizes the demographic and clinical profiles of patients in both groups. Statistical significance was assessed using chi-squared tests. Group A: COVID-19 positive; Group B: COVID-19 negative TB: tuberculosis; HIV: human immunodeficiency virus

Variable	Group A (n=100)	Group B (n=100)	P-value
Sex (male)	63 (63%)	55 (55%)	0.47
Employed	35 (35%)	38 (38%)	0.77
Past history of TB	25 (25%)	38 (38%)	0.07
Fever present	65 (65%)	70 (70%)	0.55
Cough present	56 (56%)	68 (68%)	0.11
Loss of appetite	48 (48%)	38 (38%)	0.20
Loss of weight	53 (53%)	40 (40%)	0.09
Bronchial asthma	3 (3%)	1 (1%)	0.61
Hypertension	7 (7%)	4 (4%)	0.54
HIV infection	2 (2%)	0 (0%)	0.48
Chronic kidney disease	3 (3%)	1 (1%)	0.61

**Figure 3:**
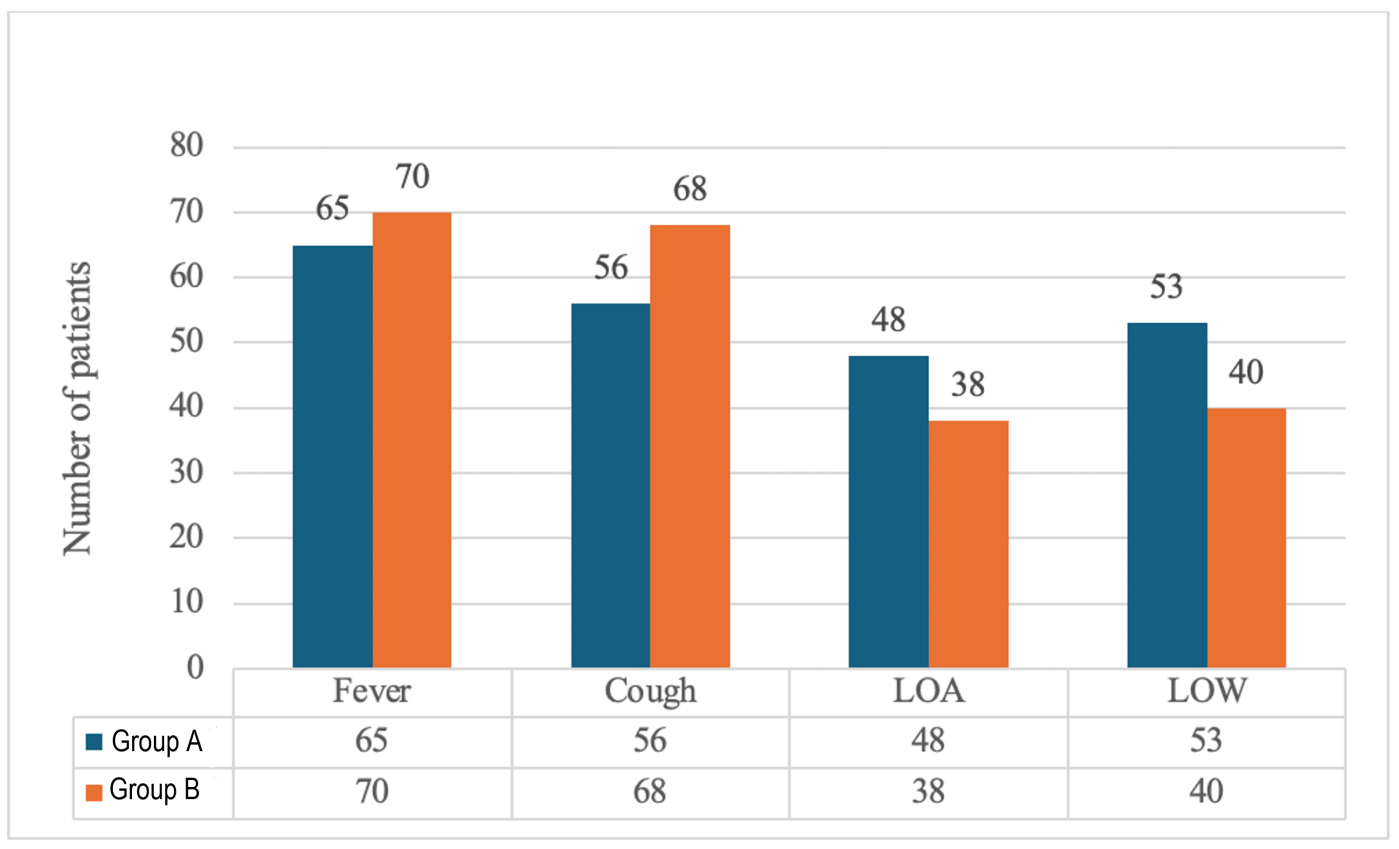
Distribution of presenting symptoms in Group A and Group B This figure compares the clinical symptoms between Group A and Group B patients. Group A: COVID-19 positive; Group B: COVID-19 negative LOA: loss of appetite; LOW: loss of weight

The distribution of major clinical symptoms among Group A and Group B participants is illustrated in Figure 3. Fever and cough were the most frequently reported symptoms in both groups. Fever was present in 65 (65%) of Group A and 70 (70%) of Group B patients, while cough was observed in 56 (56%) and 68 (68%) patients, respectively. Loss of appetite was reported by 48 (48%) patients in Group A and 38 (38%) in Group B, whereas loss of weight was observed in 53 (53%) and 40 (40%) patients, respectively. Overall, fever and cough predominated among symptomatic individuals, with slightly higher frequencies observed in the COVID-19-negative group.

Bacteriological detection of cases

Detection of *M. tuberculosis* was done by ZN staining, culture by MGIT-960, and GeneXpert® MTB/RIF assay. A comparison of the methods of detection used in the two groups is presented in Table 2. AFB were found in 12 (12%) samples from Group A and 25 (25%) samples from Group B using ZN staining. Around 19 (19%) samples from Group B and 14 (14%) samples from Group A showed culture positivity by MGIT-960, and GeneXpert® MTB/RIF assay showed comparable results between the two groups, detecting 17 (17%) cases in Group A and 19 (19%) cases in Group B. No rifampicin resistance was seen in either group. Overall, TB detection rates were lower among COVID-19 coinfected individuals by microscopy and culture, whereas molecular detection showed similar results between the two groups.

**Table 2:** Methods used for the detection of MTB Diagnostic methods including ZN staining, MGIT-960 (Becton, Dickinson and Company, Sparks, Maryland, United States), and GeneXpert® MTB/RIF assay (Cepheid, Sunnyvale, California, United States) were used. Results are expressed as numbers and percentages of total subjects in each group. Group A: COVID-19 positive; Group B: COVID-19 negative TB: tuberculosis; ZN: Ziehl-Neelsen; MGIT-960: Mycobacteria Growth Indicator Tube-960; MTB: *Mycobacterium tuberculosis*

TB detection		Group A (n=100)	Group B (n=100)
ZN staining		12 (12%)	25 (25%)
MGIT-960		14 (14%)	19 (19%)
GeneXpert MTB/RIF			
MTB detection		17 (17%)	19 (19%)
Rifampicin resistance	Detected	0	0
	Not detected	17 (17%)	19 (19%)

A phenotypic drug susceptibility test was done on culture-positive tubes. Results are shown in Table 3. 

**Table 3:** Phenotypic drug susceptibility test results for Group A and Group B This table shows the rifampicin and isoniazid susceptibility patterns across both groups using phenotypic drug susceptibility test. Group A: COVID-19 positive; Group B: COVID-19 negative

Group	Total samples tested (n)	Rifampicin sensitive, n (%)	Isoniazid sensitive, n (%)
Group A	17	17 (100%)	17 (100%)
Group B	19	19 (100%)	19 (100%)

COVID-19 vaccination history

In Group A 11 (78.5%) and in Group B 14 (73.6%) received the COVID-19 vaccination. All the cases had received two doses of the vaccine. Covishield was the predominant vaccine received in both groups. There were three patterns of sequence of COVID-19 vaccination and COVID-19 infection. The most common was having a COVID-19 infection before the COVID-19 vaccination (Table 4).

**Table 4:** Patterns of COVID-19 vaccination and sequence of COVID-19 infection observed in both groups This table compares the types of vaccines received, the doses taken, and the sequence of infection relative to vaccination in both groups. Statistical significance was calculated where applicable. Group A: COVID-19 positive; Group B: COVID-19 negative

Variable		Group A (n=100)	Group B (n=100)	P-value
COVID-19 vaccination	Covishield	64 (64%)	70 (70%)	-
	Covaxin	21 (21%)	11 (11%)	-
	None	15 (15%)	19 (19%)	-
COVID-19 vaccination	Two doses	56 (56%)	51 (51%)	-
	One dose	1 (1%)	11 (11%)	-
	None	43 (43%)	38 (38%)	-
Sequence of COVID-19 infection and vaccination	COVID-19 infection, no vaccination	43 (43%)	38 (38%)	1
	First dose, COVID-19 infection, second dose	12 (12%)	16 (16%)	
	COVID-19 infection, first dose, second dose	45 (45%)	46 (46%)	

Logistic regression analysis was conducted as shown in Table 5 with the unvaccinated group as the reference group (OR=1). In Group A, those vaccinated with Covishield had an OR of 0.57, suggesting a 43% lower odds of TB infection compared to the reference group, though this was not statistically significant (p=0.40). Covaxin recipients had a 36% lower odds (OR=0.64; p=0.58) compared to the unvaccinated individuals (reference group). Unvaccinated individuals consistently showed the highest odds of TB infection, serving as a baseline for comparison. In Group B, the odds of TB infection for Covishield were nearly unchanged (OR=0.98; p=0.98). Covaxin was associated with a 59% reduction in odds (OR=0.41; p=0.10) as compared to the unvaccinated group, though none of these were statistically significant.

**Table 5:** Logistic regression analysis comparing vaccination statuses and its association with the development of active tuberculosis Logistic regression performed using vaccination status (Covishield, Covaxin, or unvaccinated) to evaluate odds of developing tuberculosis. The unvaccinated group was used as the reference category. Group A: COVID-19 positive; Group B: COVID-19 negative

		Odds ratio	Z-value	P-value
Group A	Covishield	0.57	0.67	0.4
	Covaxin	0.64	0.8	0.58
	Unvaccinated	Reference		
Group B	Covishield	0.98	0.747	0.98
	Covaxin	0.41	0.545	0.1
	Unvaccinated	Reference		

## Discussion

The great transmissibility and lethality of COVID-19 have made it a global issue. Coinfections with COVID-19 are significant since they can make the illness potentially fatal. Given that India accounts for around 25% of global TB infections, understanding how the disease is linked to COVID-19 is crucial. Patients who had both TB and COVID-19 coinfection were more likely to experience severe illness, according to earlier research. In the majority of the individuals in our investigation, exposure to COVID-19 occurred prior to vaccination. This study explored the association between COVID-19 vaccination and the risk of TB infection among individuals with and without a history of COVID-19. The COVID-19 vaccine did not have a possible protective impact on lowering the chance of developing active TB in this study. A key strength of this study is its retrospective-prospective design, which allowed systematic data collection as well as the verification of clinical and microbiological records. In addition, the diagnostic pathway, case definition, and recruitment process were clearly outlined, enabling transparency in how active TB cases were identified.

Our study showed no statistically significant differences in most demographic and clinical variables between individuals who were COVID-19 positive (Group A) and those who were COVID-19 negative (Group B). These findings suggest that baseline characteristics such as sex, employment status, fever, cough, comorbidities, and other clinical symptoms were relatively evenly distributed across both groups. However, a near-significant trend was noted in the history of TB (p=0.07), which was more prevalent among COVID-19-negative individuals. This may reflect a protective behavioural pattern, such as reduced exposure or increased health-seeking behaviour in individuals with known TB history during the pandemic. A similar trend was also observed for weight loss (p=0.09), which was more commonly reported in the COVID-19-positive group. This could be attributed to the catabolic effects of acute viral infections, including COVID-19, which have been associated with muscle wasting and reduced appetite. Although these differences did not reach statistical significance, the trends suggest possible interactions between COVID-19 and underlying or historical TB burden. Previous studies have reported that coexisting infections and overlapping symptoms can complicate the diagnosis and management of both conditions [15,16].

The findings revealed no statistically significant protective effect of COVID-19 vaccination (Covishield or Covaxin) against TB infection in either group. In Group A, vaccinated individuals had lower odds of TB infection compared to the unvaccinated group. Although these associations did not reach statistical significance, the directionality might suggest a potential protective effect of COVID-19 vaccination against TB in individuals with concurrent SARS-CoV-2 infection. In Group B, vaccination status did not appear to significantly influence TB infection risk, and the associations were less pronounced. Covaxin was associated with a 59% reduction in TB odds (OR=0.41; p=0.10), indicating a possible protective trend, whereas Covishield showed no notable difference (OR=0.98; p=0.98) compared to the unvaccinated group. These findings, though not statistically significant, highlight the need for further investigation into vaccine-specific effects on TB susceptibility. There are several possible explanations for the observed findings. One hypothesis is that immune modulation following COVID-19 infection and vaccination may interact in unpredictable ways with the host response to *M. tuberculosis*. The concept of trained immunity, wherein certain vaccines induce the long-term functional reprogramming of innate immune cells leading to enhanced responses against subsequent infections, needs further studies [17-20]. Some studies have demonstrated that vaccines like Bacillus Calmette-Guérin (BCG) can confer non-specific protection against various pathogens through trained immunity mechanisms. Similarly, COVID-19 vaccines, particularly those utilizing adenoviral vectors like Covishield, have been shown to induce trained immunity, potentially offering cross-protection against other infections, including TB [21-23]. Furthermore, the interplay between COVID-19 and TB is complex. SARS-CoV-2 infection can modulate the immune system, potentially affecting susceptibility to other infections. Research indicates that COVID-19 can lead to long-term alterations in innate immune responses, which might influence TB reactivation or progression [24]. The observed reduction in TB infection risk among vaccinated individuals with prior COVID-19 infection may result from vaccine-induced training or the reprogramming of innate immunity, potentially mitigating the immunosuppressive effects of SARS-CoV-2 on host defense against *M. tuberculosis* [25,26].

However, this study also has important limitations. As data were collected from a single center, the findings may not be generalizable to the wider population. The restricted geographic and demographic coverage may limit external validity. Due to the observational design, the analysis is also vulnerable to possible confounding variables that were not fully controlled for, including age, comorbidities, socioeconomic factors, and previous TB exposure. Furthermore, recall bias may have been introduced because vaccination data were self-reported rather than confirmed by immunization records. Interpretation of immune or protective mechanisms is also limited by the cross-sectional design. Causality could not be confirmed from the above associations. Further, this study assessed only active TB disease and did not evaluate latent TB infection. As TST/IGRA was not performed, the prevalence of TB infection in the study population could not be determined.

Notwithstanding these drawbacks, the trends that have been noticed are significant and should be further examined by large-scale, prospective, and multicentric research. The underlying mechanisms relating to COVID-19 vaccination, trained immunity, and TB susceptibility may be clarified by future studies that include immunological and genomic evaluations. For TB-endemic areas, where integrated vaccination approaches may have major effects on pandemic preparedness and TB control, it is especially critical to understand this relationship.

## Conclusions

The current study investigated the relationship between COVID-19 vaccination and active TB in people who were categorized according to their COVID-19 status. There was a consistent trend toward lower odds of TB among vaccinated individuals compared to the unvaccinated group. Both vaccines demonstrated a directionally protective effect against TB in the COVID-19-positive group, indicating a possible potential immunomodulatory function of COVID-19 vaccines. The results do not establish causality due to the small sample size. Further multi-center, longitudinal studies with larger sample sizes are required to confirm these findings and to better understand the potential interaction between COVID-19 vaccination, COVID-19 infection, and active TB disease.
